# Mitigation of Sodium Iodate-Induced Cytotoxicity in Retinal Pigment Epithelial Cells *in vitro* by Transgenic Erythropoietin-Expressing Mesenchymal Stem Cells

**DOI:** 10.3389/fcell.2021.652065

**Published:** 2021-04-15

**Authors:** Avin Ee-Hwan Koh, Suresh Kumar Subbiah, Aisha Farhana, Mohammad Khursheed Alam, Pooi Ling Mok

**Affiliations:** ^1^Department of Clinical Laboratory Sciences, College of Applied Medical Sciences, Jouf University, Sakaka, Saudi Arabia; ^2^Department of Biomedical Sciences, Faculty of Medicine and Health Sciences, Universiti Putra Malaysia, Seri Kembangan, Malaysia; ^3^Department of Medical Microbiology, Universiti Putra Malaysia, Seri Kembangan, Malaysia; ^4^Genetics and Regenerative Medicine Research Group, Universiti Putra Malaysia, UPM, Seri Kembangan, Malaysia; ^5^Centre for Materials Engineering and Regenerative Medicine, Bharath Institute of Higher Education and Research, Chennai, India; ^6^Department of Orthodontics, College of Dentistry, Jouf University, Sakaka, Saudi Arabia

**Keywords:** mesenchymal stem cells, erythropoietin, sodium iodate, retinal pigment epithelium, cell death

## Abstract

Mesenchymal stem cells (MSC) have shown promise in restoring the vision of patients in clinical trials. However, this therapeutic effect is not observed in every treated patient and is possibly due to the inefficacies of cell delivery and high cell death following transplantation. Utilizing erythropoietin can significantly enhance the regenerative properties of MSCs and hence improve retinal neuron survivability in oxidative stress. Hence, this study aimed to investigate the efficacy of conditioned medium (CM) obtained from transgenic human erythropoietin-expressing MSCs (MSC_*EPO*_) in protecting human retinal pigment epithelial cells from sodium iodate (NaIO_3_)-induced cell death. Human MSC and MSC_*EPO*_ were first cultured to obtain conditioned media (CM). The IC_50_ of NaIO_3_ in the ARPE-19 culture was then determined by an MTT assay. After that, the efficacy of both MSC-CM and MSC-CM_*EPO*_ in ARPE-19 cell survival were compared at 24 and 48 h after NaIO_3_ treatment with MTT. The treatment effects on mitochondrial membrane potential was then measured by a JC-1 flow cytometric assay. The MTT results indicated a corresponding increase in cell survivability (5–58%) in the ARPE-19 cell cultures. In comparison to MSC-CM, the use of conditioned medium collected from the MSC-CM_*EPO*_ further enhanced the rate of ARPE-19 survivability at 24 h (*P* < 0.05) and 48 h (*P* < 0.05) in the presence of NaIO_3_. Furthermore, more than 90% were found viable with the JC-1 assay after MSC-CM_*EPO*_ treatment, showing a positive implication on the mitochondrial dynamics of ARPE-19. The MSC-CM_*EPO*_ provided an enhanced mitigating effect against NaIO_3_-induced ARPE-19 cell death over that of MSC-CM alone during the early phase of the treatment, and it may act as a future therapy in treating retinal degenerative diseases.

## Introduction

The application of stem cells in treating retinal degenerative diseases is a growing niche in the regenerative medicine field (Ng et al., 2014; Tang et al., 2017). Over the years, different types of stem cells have been used in various transplant procedures, including embryonic stem cells (Schwartz et al., 2012, 2015) and retinal progenitor cells (Liu et al., 2017). Mesenchymal stem cells (MSC), in particular, have huge potential as a cellular therapeutic agent. This is in regards to its ease of procurement (Ng et al., 2014), non-controversial ethical use (Tang et al., 2017), and most importantly, the cells’ ability to differentiate and repair wounded tissue (Wang et al., 2014). These cells can be harvested from the umbilical cord, bone marrow, dental pulp, adipose tissue, etc., without inflicting grievous harm to the donors (Hass et al., 2011). MSCs are also capable of mediating the immune response and promoting tissue regeneration through the release of paracrine effectors (Berglund et al., 2017; Saldaña et al., 2017). To date, there are numerous ongoing clinical trials that are exploring the possible benefits of MSC transplants in treating several eye disorders (e.g., NCT02330978 and NCT02016508). However, the employment of transgenic stem cells in this particular field of study is relatively new. Such a method has previously been used in other cases, for example, to regenerate a patient’s skin using epidermal stem cells that have been engineered to express laminin (Bauer et al., 2017; Hirsch et al., 2017). This two-pronged approach is thought to elicit a better therapeutic effect.

Erythropoietin (EPO) is the principal hormone required for the body to produce red blood cells. It is also known to possess several non-hematopoietic functions (Ding et al., 2016), most notably in promoting anti-apoptotic property (Lombardero et al., 2011) and neurogenesis (Sasaki et al., 2000). This hormone has been implicated in human fetal eye development, and the expression of EPO receptors in the human retina (Patel et al., 2008), including other mammals such as mice (Caprara et al., 2014) and rats (Xie et al., 2007), are well-retained after birth (Ding et al., 2016). Additionally, it is believed that EPO is capable of modulating the inflammatory response within the eye by reducing the levels of reactive nitrogen/oxygen species (RNS/ROS) (Bond and Rex, 2014), and suppressing the expression of pro-inflammatory cytokines, e.g., tumor necrosis factor-alpha (TNF-α) and interleukin-6 (IL-6) (Luo et al., 2015). Such inflammation is common in eye disorders, including retinitis pigmentosa (RP) (Hanus et al., 2016) and age-related macular degeneration (AMD) (Kauppinen et al., 2016).

In a normal physiological environment, the exposure of photoreceptors to light rays results in a harmful oxidative environment that is neutralized by the RPE to ensure the survival of both tissues (Sparrow et al., 2010). However, a dysfunction in the RPE will lead to the build-up and consequently, death of photoreceptors from oxidative stress (Kevany and Palczewski, 2010). Currently available treatment aimed to alleviate such tissue loss, as observed in RP and AMD, is such as recombinant anti-vascular endothelial growth factor (VEGF) therapy (Jo et al., 2014). More recently, researchers are exploring the application of genetically modified MSCs to enhance graft survival and cellular reparative mechanisms to recover lost retinal tissue. In our previous study, we have successfully demonstrated that cytotoxicity of glutamic acid on Y79 retinal cell line of photoreceptor origin could be significantly reduced by exposure of cells to conditioned medium collected from recombinant EPO-expressing MSCs (MSC-CM_*EPO*_) (Ding S. L. et al., 2018). Hence, we hypothesized that MSC-CM_*EPO*_ could also have the same rescue effect on RPE cells undergoing necroptosis. Necroptosis is a cell death mechanism that contributes to retinal degeneration in known ocular disorders such as AMD (Hanus et al., 2016). To elucidate this, we exposed ARPE-19 cell lines to sodium iodate and then incubated the cells with MSC-CM_*EPO*_. The survivability of ARPE-19 cells was then assessed and compared with that of conditioned media collected from unmodified cells (MSC-CM) using colorimetric and flow cytometric analysis.

## Materials and Methods

### Cell Culture of ARPE-19, MSC, and MSC_*EPO*_

The human RPE cell line ARPE-19 (ATCC, Virginia, United States) were cultured from P3 onwards in complete culture medium consisting of DMEM/F12 (Thermo Fisher Scientific, Massachusetts, United States), 10% fetal bovine serum (FBS) (Thermo Fisher Scientific, Massachusetts, United States), and 1% penicillin-streptomycin (Thermo Fisher Scientific, Massachusetts, United States). A short-tandem-repeat (STR) profiling for ARPE-19 authentication was performed by matching twenty-two STR loci plus the gender determining locus, Amelogenin and male-specific DYS391 locus of ARPE-19 against the reference ARPE-19 (ATCC^®^ CRL-2302^TM^) STR profile. The loci were D3S1358, D1S1656, D2S441, D10S1248, D13S317, Penta E, D16S539, D18S51, D2S1338, CSF1PO, Penta D, TH01, vWA, D21S11, D7S820, D5S818, TPOX91, DYS391, D8S1179, D12S391, D19S433, FGA, D22S1045. The sample was processed using the ABI PRISM^®^ 3100 Genetic Analyzer, and the resulting data was analyzed using the GeneMapper^®^ v5.0 software (Applied Biosystems^TM^). The percent match was 100% (Supplementary Figure 1). The human Wharton’s Jelly-derived MSCs were kindly donated by Cryocord Sdn. Bhd. (Cyberjaya, Selangor, Malaysia), and cultured from P1 onwards in complete culture medium consisting of DMEM/F12, 10% FBS, and 1% penicillin-streptomycin. Upon reaching 70% confluency, the respective cells were passaged by trypsinization using 0.25% trypsin-EDTA (Thermo Fisher Scientific, Massachusetts, United States) and centrifugation at 1,000× *g.* MSCs_*EPO*_ were generated from the MSCs according to the protocol outlined in our previous study by Ding S. L. et al. (2018). In brief, recombinant lentiviral particles containing the pReceiver-Lv183-EPO plasmid (NCBI accession number: NM_000799.2) (GeneCopoeia, Maryland, United States) were added into the MSC (P3 to P6) complete culture medium supplemented with 8 μg/ml of polybrene (Sigma-Aldrich, Missouri, United States). After an 8 h incubation, the viral particles were removed and fresh complete growth culture medium was added. The transduced cells (MSC_*EPO*_) were then culture expanded. Flow cytometry was used to assess transduction efficiency, which was determined at 11.9%, followed by cell sorting (Ding et al., 2019). The pReceiver-Lv183-EPO plasmid comes with an upstream CMV promoter and a *GFP* expression tag downstream of *EPO*. It also contains neomycin and ampicillin as selection markers. All cell cultures were maintained under standard culture conditions (5% CO_2_ at 37°C in a humidified cell culture incubator).

### Determination of EPO Secretion From the Transduced Cells

The transduced cells were culture-expanded for three sub passages before being sorted by a flow cytometer (Ding et al., 2019). The sorted cells were used in the present study. The secretion of EPO was determined by using an EPO ELISA kit according to the product specification (eBioscience, California, United States). The procedures to measure the EPO secretion have been described previously in Ding S. L. et al. (2018).

### IC_50_ Determination of Sodium Iodate

Sodium iodate powder (NaIO_3_) (Alfa Aesar, Massachusetts, United States) was dissolved in DMEM/F12 media to obtain a stock solution of 100 mM, and filter-sterilized using a 0.22 μm polyethersulfone syringe filter (Nanogene, Selangor, Malaysia). Cellular toxicity in ARPE-19 cells was induced in a 24-well plate by spiking 0–40 mM of NaIO_3_ (*n* = 9 per dose) into the culture medium. The induced cultures were observed under a phase-contrast microscope throughout the study. After 48 h, the cell viability was then analyzed using a 3-(4,5-dimethylthiazol-2-yl)-2,5-diphenyltetrazolium bromide (MTT) assay. In brief, the NaIO_3_-treated ARPE-19 cells were incubated with 0.05 mg/ml of MTT reagent (Sigma-Aldrich, Missouri, United States) for 4 h. After that, the media was discarded and dimethyl sulfoxide (DMSO) was added into the wells to dissolve the formazan crystals. The solution was then transferred to a 96-well plate for optical density (OD) measurement using an absorbance wavelength of 570 nm. The reference wavelength of 630 nm was used. The IC_50_ was determined by analyzing the dose-response data using the four parameter logistic model (Sebaugh, 2011).

### Determination of Survivability of ARPE-19 Cells Following Exposure to NaIO_3_ and Treatment With MSC-CM and MSC-CM_*EPO*_

Before preparing the CM, ARPE-19 cells were seeded at 3,000 cells/cm^2^ and incubated until about 50% confluency. Then, the media was refreshed and NaIO_3_ was added (6.8 mM final concentration). After a 24 h incubation, the media was removed. Following that, the NaIO_3_-induced ARPE-19 cell cultures were added with either 50% of MSC-CM or MSC-CM_*EPO*_ in complete culture medium. The cells were incubated for 48 h under standard culture conditions. The conditioned media were collected from MSCs (MSC-CM) and MSC_*EPO*_ (MSC-CM_*EPO*_) cultures. The description was as follows. The cells were first grown in complete culture medium for 48 h until reaching about 60% confluency. After that, the medium was changed to a serum-free version of the culture medium and the cells were incubated for another 48 h. The conditioned media were then collected and centrifuged at 1,000 × *g* for 10 min. The supernatant was extracted to yield a stock volume of conditioned media. After the incubation the cell viabilities were then determined by using an MTT assay according to the procedure stated above and compared with that of a sham treatment (without the addition of conditioned media).

### Determination of ARPE-19 Mitochondrial Membrane Potential (ΔΨm) Changes Following Stem Cell CM Treatment

The ARPE-19 ΔΨm was measured using a JC-1 Flow Cytometry assay kit (Cayman Chemical, Czech Republic, Germany) according to the provided protocol. After treatment with conditioned medium according to the methods stated in above paragraph at 48 h, ARPE-19 cells were trypsinized and centrifuged under standard culture methods, and washed with PBS (pH 7.4) for up to three times. The cells were resuspended and incubated in 100 μl assay buffer for 5 min, and then added with an equal volume of 100 μl JC-1 staining solution. The samples were analyzed using a BD FACSCanto II flow cytometer with 488 and 525 nm channels.

### Statistical Analysis

One and two-way analysis of variance (ANOVA) together with Tukey’s multiple comparison test *post-hoc* were used to compare the mean differences between each respective treatment groups (*n* >3 per dose) in the study. For the flow cytometric analysis, a one-way ANOVA was performed followed by Tukey’s multiple comparison test (*n* = 3). Significance was defined as *p* <0.05.

## Results

### Cytotoxicity of NaIO_3_ Toward ARPE-19

**FIGURE 1:**
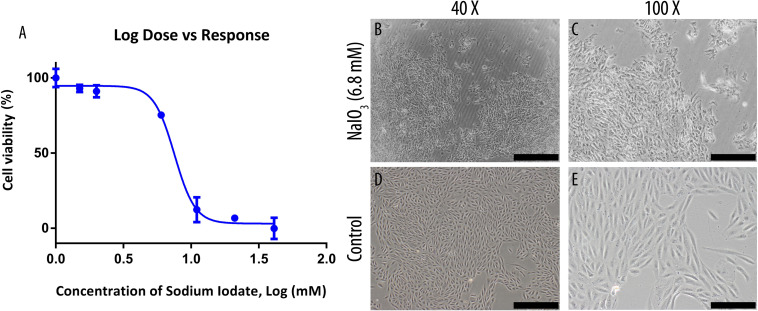
Sodium iodate toxicity on human ARPE-19 cell line was determined by MTT assay and its effects on cell morphology were observed under a bright-field microscope. **(A)** The IC_50_ of the compound was determined through an MTT assay and was found to be 6.8 mM. The data were shown as mean ± SEM, *n* = 9. **(B,C)** ARPE-19 cells were cultured in serum-supplemented basal media and 6.8 mM NaIO_3_. **(D,E)** ARPE-19 cells were cultured in serum-supplemented basal media without NaIO_3_ (control) treatment. The scale bars denote 500 and 200 μm at 40× and ×100 total magnification, respectively.

The inhibitory concentration (IC_50_) of NaIO_3_ on the growth of ARPE-19 cells were determined by MTT assay following exposure to 0–40 mM of NaIO_3_ (Figure 1A). The cell viabilities reduced drastically when incubated in 5–40 mM concentration, resulting in a sigmoid curve that plateaus from 10 mM onwards at near 0% viability. Using GraphPad Prism 6, the IC_50_ was determined to be 6.8 mM. At this concentration, the cells exhibited losses of cell-to-cell adhesion, and shrinkage in cell size (Figures 1C,E). Meanwhile, untreated ARPE-19 cells demonstrated normal epithelial morphology, which is characterized by the formation of a monolayer of highly regular polygonal-shaped cells (Figures 1B,D).

### Determination of EPO Secretion From the Transduced MSCs

**FIGURE 2:**
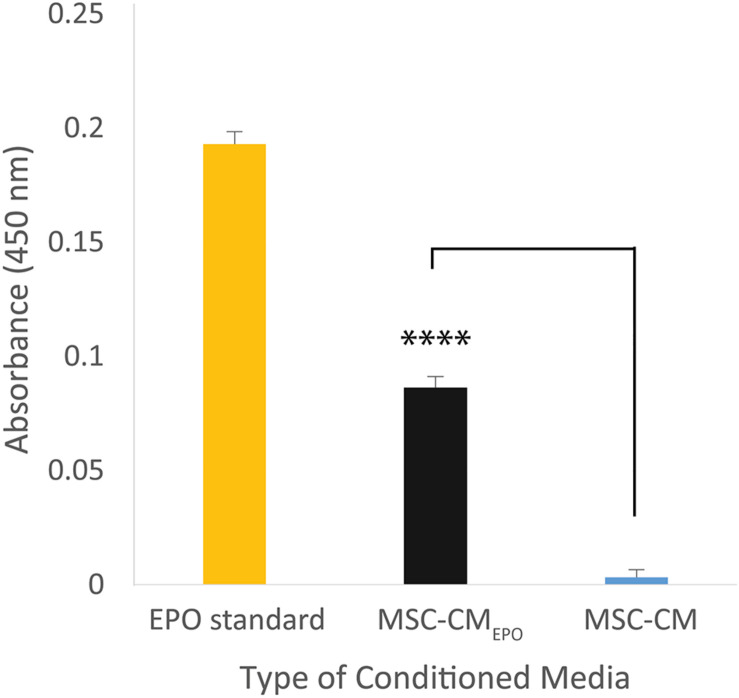
The amount of erythropoietin secreted by MSC_*EPO*_ in the conditioned medium was quantified using a sandwich ELISA assay. The cells were seeded into a 6-well plate to achieve a seeding density of approximately 30 000 cells/cm^2^, *n* = 3. Once 80% confluency was achieved, the media was refreshed and the cells were incubated for 24 h under standard culture conditions (37°C, 5% CO_2_). The media was then collected for ELISA assay, and the amount of EPO in MSC-CM_*EPO*_ conditioned medium was found to be 1 mIU/ml. The Student’s *T*-test was used to calculate the significant difference between the MSC-CM_*EPO*_ and MSC-CM group. ^*⁣*⁣**^p < 0.0001.

The secretion of EPO in the MSC-CM_*EPO*_ conditioned medium was validated by measuring its amount using an ELISA kit (Figure 2). Compared to the non-transduced cells, the transduced MSCs showed presence of EPO in the collected medium. The amount of EPO in MSC-CM_*EPO*_ conditioned medium was found to be 1 mIU/ml.

### Dose-Dependent Effect of Conditioned Media on Treated ARPE-19 and Enhanced Survivability With MSC-CM_*EPO*_

**FIGURE 3:**
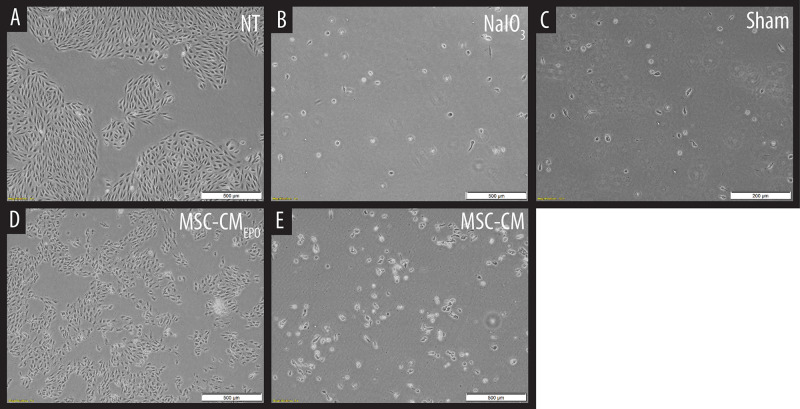
Observation of sodium iodate-induced cell death of ARPE-19 cells in MSC-CM and MSC-CM_*EPO*_ conditioned medium over a 48 h period. **(A)** Non-treated (NT) culture of ARPE-19 cells. **(B)** ARPE-19 cells induced with sodium iodate. **(C)** Sodium iodate-induced ARPE-19 cells treated with a sham control. **(D)** Sodium iodate-induced ARPE-19 cells were treated with MSC-CM_*EPO*_ conditioned medium. **(E)** Sodium iodate-induced ARPE-19 cells were treated with MSC-CM conditioned medium. The treated cultures were observed under a brightfield microscope at 40× total magnification, and representative images were captured. The scale bars denote 500 μm.

**FIGURE 4:**
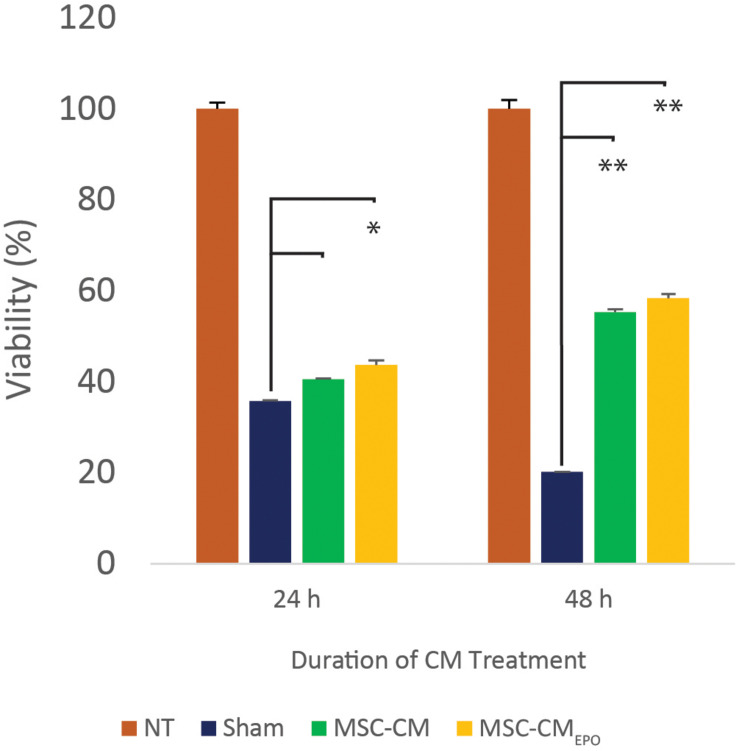
The mitigating effect of MSC-CM and MSC-CM_*EPO*_ on sodium iodate-induced cell death of ARPE-19 cells over a 24 and 48 h period. The effects of 50% MSC-CM and MSC-CM_*EPO*_ on ARPE-19 survivability were measured using an MTT assay and the data were shown as mean ± SEM, *n* = 4. Two-way ANOVA was used to calculate the significant difference, followed by Tukey’s multiple comparisons test where measurements were compared using sham as the reference. ^∗^*p* < 0.001, ^∗∗^*p* < 0.0001.

**FIGURE 5:**
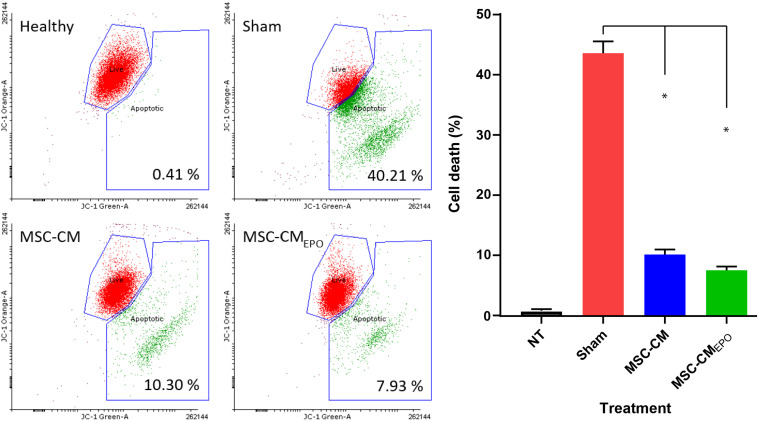
The positive effects of stem cell conditioned medium on ARPE-19 survivability following addition of NaIO_3_. ARPE cells were added with NaIO_3_ and treated with MSC-CM or MSC-CM_*EPO*_. The viability at 48 h was then measured by determining the change in mitochondrial membrane potential (ΔΨm) of ARPE-19 cells. Two control tests were also performed. One control group contains ARPE cells alone without addition of NaIO_3_ and stem cell CM. Meanwhile, sham control group was induced with NaIO_3_ and treated with PBS only. Live cells were labeled as green dots while early apoptotic cells were labeled red. The JC-1 stains were detected by both 525 nm (JC-1 orange) and 488 nm (JC-1 green) channels, respectively. The results were presented as mean ± SEM, *n* = 3. One-way ANOVA was used to determine statistical significance between sham and the treated groups. ^∗^*p* < 0.0001. NT, no-treatment.

The viability of ARPE-19 in NaIO_3_-treated cultures was then observed under a brightfield microscope and assessed by MTT assay after the addition of MSC-CM or MSC-CM_*EPO*_. The MSC-CM_*EPO*_-treated cells exhibited a morphology that was very similar to the untreated cells, which grew as regular patches of epithelia. The MSC-CM-treated cells also retained a similar morphology; however, the cells were sparse compared to the MSC-CM_*EPO*_ group (Figure 3). At 24 h, the MSC-CM was not able to revive the ARPE-19 cells against oxidative damage by NaIO_3_. A statistically significant rescue (*p* < 0.0001) was only achievable after 48 h. Meanwhile, MSC-CM_*EPO*_ could increase the cell viability significantly at 24 h (*p* < 0.001) and 48 h (*p* < 0.0001) in comparison to the control (Figure 4). NaIO_3_ induces mitochondrial depolarization in ARPE-19 cells. When treated with its IC_50_ and a vehicle (sham), approximately 40% of the cells experienced early apoptosis. Addition of MSC-CM and MSC-CM_*EPO*_ into the NaIO_3_-ARPE cell culture reduced cell death to 10.30 and 7.93%, respectively (Figure 5).

## Discussion

In this study, the NaIO_3_-induced RPE degeneration model was used to assess the treatment efficacy of MSC-CM_*EPO*_ and MSC-CM. The compound is a cytotoxic agent that causes RPE necroptosis by inducing aggregation of receptor-interacting serine/threonine-protein kinase 3 (RIPK3), which triggers a form of cell death known as necroptosis (Hanus et al., 2016). It is also possible that cell death may occur by induction of oxidative stress (Sachdeva et al., 2014). Previous study have shown that it can react specifically with this cell type in many animal species [e.g., and rabbits (Shen et al., 2010), rats (Alsaeedi et al., 2019), and sheep (Nilsson et al., 1977)]. When added into the ARPE-19 cell culture, a loss of epithelial cell morphology could be observed within 24 h (Figure 1). The current *in vitro* model can be used to represent age-related macular degeneration, which is an incurable ocular disorder that is characterized by the loss of RPE cells (Fernández-Robredo et al., 2014). In order to prevent further loss of RPE cells, MSCs can be used as a cell source for treatment (Cislo-Pakuluk and Marycz, 2017). Recently, the production of synthetic MSCs through the packaging of MSC-secreted factors into carriers have shown high promise in animal models of osteoporosis (Shen et al., 2018) and acute myocardial infarction (Lee et al., 2017). This method replaces the direct use of MSC in treatment with the secretome, and thus, circumventing certain drawbacks such as reduced transplant survivability (English and Wood, 2013), undesired differentiation (Volarevic et al., 2018), and tumor promotion (Volarevic et al., 2018).

These MSCs are able to release extracellular vesicles (i.e., exosomes and microvesicles) and secrete soluble factors that promote tissue regeneration (Baraniak and McDevitt, 2010). A large number of such factors were previously identified, including but not limited to, vascular endothelial growth factor (VEGF), transforming growth factor beta 1 (TGFβ1), nerve growth factor (NGF), and insulin-like growth factor-1 (IGF1), etc. (Baraniak and McDevitt, 2010). In addition to that, the vesicles are a host to non-coding RNA species such as miRNA (Fatima et al., 2017; Ferguson et al., 2018). These have been found to target pathways of proliferation and apoptosis, such as the Wnt signaling pathway. Several studies have correlated the improvement of tissue repair with the use of MSC-CM. In an animal study, Jiang et al. (2017) were able to promote the healing of chemically injured corneal epithelium in a mouse model using MSC-CM (Jiang et al., 2017). While in a clinical study, Dahbour et al. (2017) found an improvement in patients with multiple sclerosis after combining bone marrow-MSCs and MSC-CM in their treatment regime (Dahbour et al., 2017). By using this treatment, they found a reduction in symptoms and overall improvement in prognosis. In our previous study, we have characterized the Wharton’s Jelly-derived MSCs by differentiation into the characteristic mesodermal lineages as well as validation of CD90, CD73, CD105, CD29, CD44, and HLA-ABC expression (Ding S. L. et al., 2018). Furthermore, we have transduced EPO gene into these cells and showed that exposure of conditioning medium harvested from these cells had a magnifying rescue effect over unmodified cells on the degeneration of retinal neurons due to oxidative stress (Ding S. L. et al., 2018). Hence, in the current study, we further tested the efficiency of the conditioning medium containing EPO protein for its ability to promote cell survivability in ARPE cell line when exposed to the cytotoxic agent.

Prior to our experiments, the secretion of EPO in the human MSC_*EPO*_ cell culture was confirmed again by ELISA (Figure 2). Our results indicated that the collected medium from these cells was able to significantly enhance ARPE-19 survival than MSC-CM within 24 h post-treatment (*p* < 0.001) (Figure 4). At 48 h, the MSC-CM treatment cell group was found capable of exhibiting equal rescue effect of MSC_*EPO*_-CM. Meanwhile, the treatment of MSC_*EPO*_-CM showed a higher positive implication on the mitochondrial dynamics of ARPE-19, where more than 90% of the cells were found viable at 48 h (Figure 5). Both treatment groups exposed to MSC_*EPO*_-CM and MSC-CM were not showing significant difference in the number of apoptotic cells in the mitochondrial depolarization assay (Figure 5), which corresponded to our MTT cell viability assay. Taken together, our results indicated the potential of EPO protein to enhance the intrinsic regenerative potential in MSC in the early phase of treatment. The effect was not observed in the later 48 h, probably due to its low concentration. Our preliminary results may show potential significance in a clinical setting, in which immediate treatment is vital to reduce further damage to RPE cells, thereby preventing severity in visual dysfunction progression. Our results also point to the need to employ a higher EPO concentration for treating RPE cells when compared to neural photoreceptors.

The main limitation in the present study was that the amount of EPO in MSC_*EPO*_-CM was limited. The transduction protocol needs further optimization to improve the secretion of EPO by the cells. Modifying the protocol to a co-culture experiment with MSC_*EPO*_ and ARPE-19 cells would likely have produced a much more significant result by the continuous supply of EPO, hence, eliciting a constant pro-survival effect. Also, we recommend changing the ARPE-19 cell line to a more physiologically relevant RPE cells, such as primary RPE culture or induced pluripotent stem cell (iPSC)-derived RPE cells, to generate a more accurate result in the near future (Poon et al., 2015; Zhang et al., 2020b). In the present study, Wharton’s jelly MSC was used because these cells boast enhanced proliferative markers and immunomodulatory capabilities (Kamal and Kassem, 2020). However, the use of Wharton’s jelly MSC may also lead to inconsistent results as these postnatal cells are prone to donor-specific variations, such as increased senescence, that can limit its therapeutic efficacy (Zhang et al., 2020a). Using other cell types, such as iPSC-derived MSCs have been shown to have enhanced proliferation as well as safety in human trials (Zhang et al., 2012; Lian and Zhang, 2016; Bloor et al., 2020).

EPO may have promoted cell survivability by activation of NF-κB (Li et al., 2004), phosphoinositide 3-kinase (PI3-K) (Günter et al., 2013), or protein kinase B (Akt) (Wang et al., 2009) which antagonized the damage signals brought about by NaIO_3_. Indirectly, the hormone may have also initiated cell survival activity via the stimulation of effectors such as hypoxia-inducible factors alpha (HIF-1α) and inhibition of pro-apoptotic factors such as caspase-3 (Güven Bağla et al., 2018). The application of EPO on ROS-induced RPE cytotoxicity *in vitro* have been shown to provide protective effects (Wang et al., 2009). Not only that, the EPO was also shown to act on the MSC itself by preserving stemness (Güven Bağla et al., 2018) and even protecting the cells from hyperglycemic injury (Cui et al., 2019). Furthermore, the application of rat MSCs_*EPO*_ against retinal degeneration were shown to be effective *in vivo* (Guan et al., 2013). On the other hand, EPO was also found to activate the RAP1 signaling pathway (Arai et al., 2001), which also involved the regulation of NF-κB. Downregulation of NF-κB could lead to reduction of paracrine secretion from the MSCs (Zhang et al., 2015; Ding Y. et al., 2018), and this might be responsible for the reduced cell survivability percentage at 48 h. Therefore, it warrants further investigation on how this interaction will affect the treatment efficiency of MSC_*EPO*_ in the future. More investigations on the involving signaling pathways to reveal the molecular events in ARPE cells upon interaction with EPO protein needs to be done too.

## Conclusion

In brief, sodium iodate is an oxidative agent that results in ARPE-19 cell death. The study showed that MSC-CM_*EPO*_ was able to significantly improve the viability of ARPE-19 upon the induction of cell death by sodium iodate_3_ in the early treatment phase. The mitigation in cell death was comparably higher than that of MSC-CM. The improvement in early treatment efficacy is likely due to synergistic effects between EPO and paracrine effectors in MSC-CM_*EPO*_. The current study has demonstrated a proof-of-concept where conditioned medium harvested from erythropoietin-expressing mesenchymal stem cells can be used to rescue retinal pigment epithelial cell death.

## Data Availability Statement

All datasets generated for this study are included in the article/Supplementary Material, further inquiries can be directed to the corresponding author/s.

## Author Contributions

PLM was responsible for conceiving the experimental study design, analyzing the data, and editing the manuscript. AE-HK performed the experiments and composed the manuscript. SKS supported the study design and analyzed all the data. AF and MKA analyzed and edited the manuscript. All authors were involved in reviewing the manuscript.

## Conflict of Interest

The authors declare that the research was conducted in the absence of any commercial or financial relationships that could be construed as a potential conflict of interest.

## Supplementary Material

Supplementary Figure 1 Short-tandem-repeat (STR) profiling analysis of ARPE-19. Eight of the tested loci, D13S317, D16S539, D18S51, CSF1PO, TH01, vWA, D7S820, D5S818, and TPOX, were matched with the ARPE-19 (ATCC^®^ CRL-2302^TM^) reference STR profile, and had 100% similarity. The sample was processed using the ABI PRISM^®^ 3100 Genetic Analyzer, and the resulting data was analyzed using the GeneMapper^®^ v5.0 software (Applied Biosystems^TM^).
