# Corrigendum: Mitigation of Sodium Iodate-Induced Cytotoxicity in Retinal Pigment Epithelial Cells *in vitro* by Transgenic Erythropoietin-Expressing Mesenchymal Stem Cells

**DOI:** 10.3389/fcell.2021.770837

**Published:** 2021-10-04

**Authors:** Avin Ee-Hwan Koh, Suresh Kumar Subbiah, Aisha Farhana, Mohammad Khursheed Alam, Pooi Ling Mok

**Affiliations:** ^1^Department of Clinical Laboratory Sciences, College of Applied Medical Sciences, Jouf University, Sakaka, Saudi Arabia; ^2^Department of Biomedical Sciences, Faculty of Medicine and Health Sciences, Universiti Putra Malaysia, Seri Kembangan, Malaysia; ^3^Department of Medical Microbiology, Universiti Putra Malaysia, Seri Kembangan, Malaysia; ^4^Genetics and Regenerative Medicine Research Group, Universiti Putra Malaysia, UPM, Seri Kembangan, Malaysia; ^5^Centre for Materials Engineering and Regenerative Medicine, Bharath Institute of Higher Education and Research, Chennai, India; ^6^Department of Orthodontics, College of Dentistry, Jouf University, Sakaka, Saudi Arabia

**Keywords:** mesenchymal stem cells, erythropoietin, sodium iodate, retinal pigment epithelium, cell death

In the published article, there was an error in the affiliations.

Instead of

“Department of Medical Microbiology and Parasitology, Universiti Putra Malaysia, UPM, Seri Kembangan, Malaysia” it should be “Department of Medical Microbiology, Universiti Putra Malaysia, Seri Kembangan, Malaysia.”

And

“Department of Biotechnology, Bharath Institute of Higher Education and Research, Chennai, India” should be “Centre for Materials Engineering and Regenerative Medicine, Bharath Institute of Higher Education and Research, Chennai, India.”

In the published article, there was an error regarding the affiliations for Avin Ee-Hwan Koh. They have two affiliations, which are “Department of Clinical Laboratory Sciences, College of Applied Medical Sciences, Jouf University, Sakaka, Saudi Arabia.” And “Department of Biomedical Sciences, Faculty of Medicine and Health Sciences, Universiti Putra Malaysia, Seri Kembangan, Malaysia.”

The authors apologize for this error and state that this does not change the scientific conclusions of the article in any way. The original article has been updated.

## Publisher's Note

All claims expressed in this article are solely those of the authors and do not necessarily represent those of their affiliated organizations, or those of the publisher, the editors and the reviewers. Any product that may be evaluated in this article, or claim that may be made by its manufacturer, is not guaranteed or endorsed by the publisher.

