# Supplementary figures and images for: Mitigation of Sodium Iodate-Induced Cytotoxicity in Retinal Pigment Epithelial Cells in vitro by Transgenic Erythropoietin-Expressing Mesenchymal Stem Cells
Source: Front Cell Dev Biol. 2021 Apr 15;9:652065. doi: 10.3389/fcell.2021.652065 (PMC8082501; doi:10.3389/fcell.2021.652065)

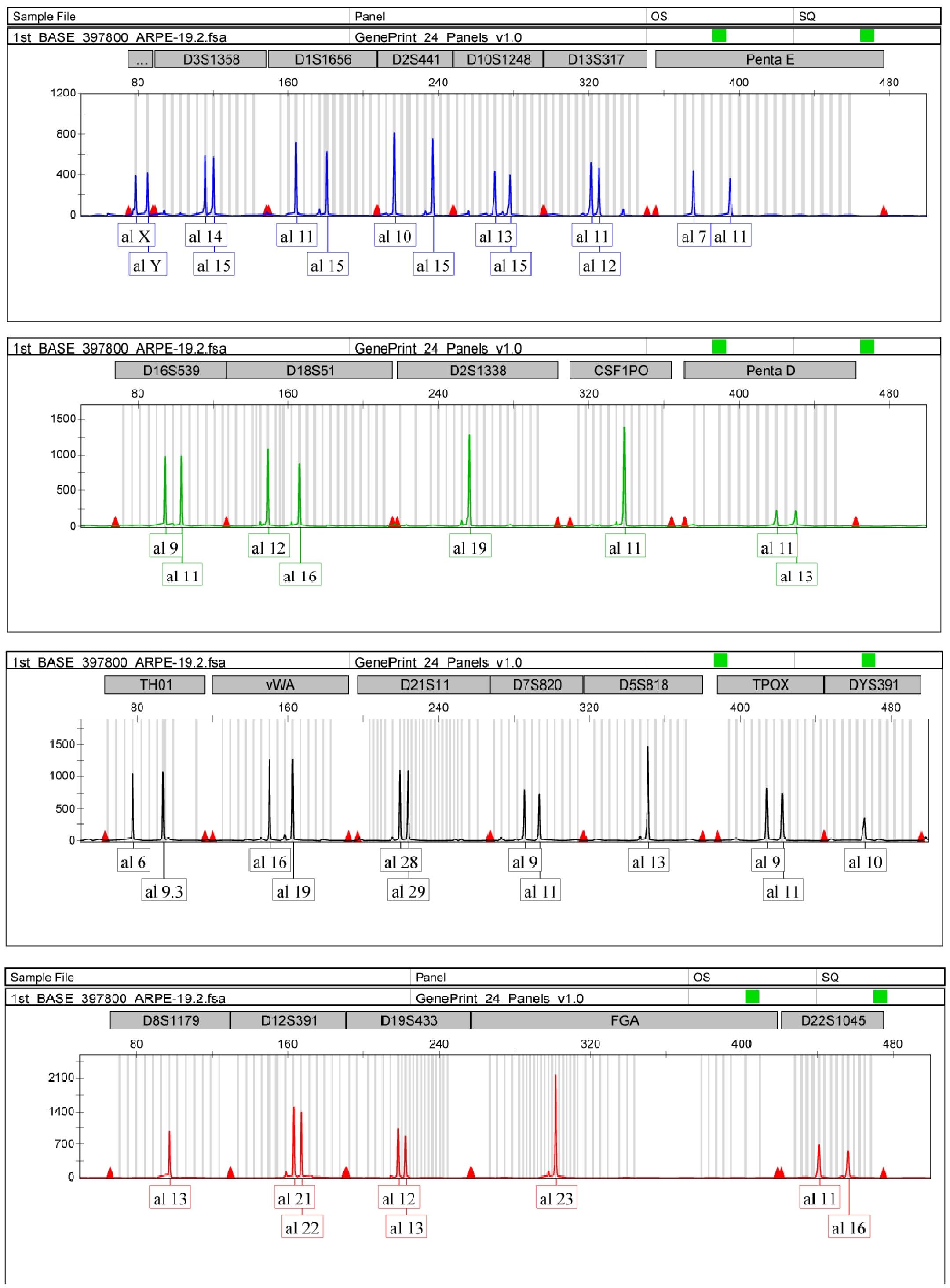

Supplement: Supplementary Figure 1 — Short-tandem-repeat (STR) profiling analysis of ARPE-19. Eight of the tested loci, D13S317, D16S539, D18S51, CSF1PO, TH01, vWA, D7S820, D5S818, and TPOX, were matched with the ARPE-19 (ATCC® CRL-2302TM) reference STR profile, and had 100% similarity. The sample was processed using the ABI PRISM® 3100 Genetic Analyzer, and the resulting data was analyzed using the GeneMapper® v5.0 software (Applied BiosystemsTM). [file Image_1.JPEG]
